# Inflammatory retiform purpura and wrist drop

**DOI:** 10.1016/j.jdcr.2025.03.018

**Published:** 2025-04-01

**Authors:** Mason Seely, Milbrey Parke, Kiran Motaparthi

**Affiliations:** a Department of Internal Medicine, University of Florida College of Medicine, Gainesville, Florioda; b Department of Dermatology, University of Florida College of Medicine, Gainesville, Florida

**Keywords:** medium vessel vasculitis, mononeuritis multiplex, retiform purpura, rheumatoid vasculitis

## Clinical history

**Fig 1:**
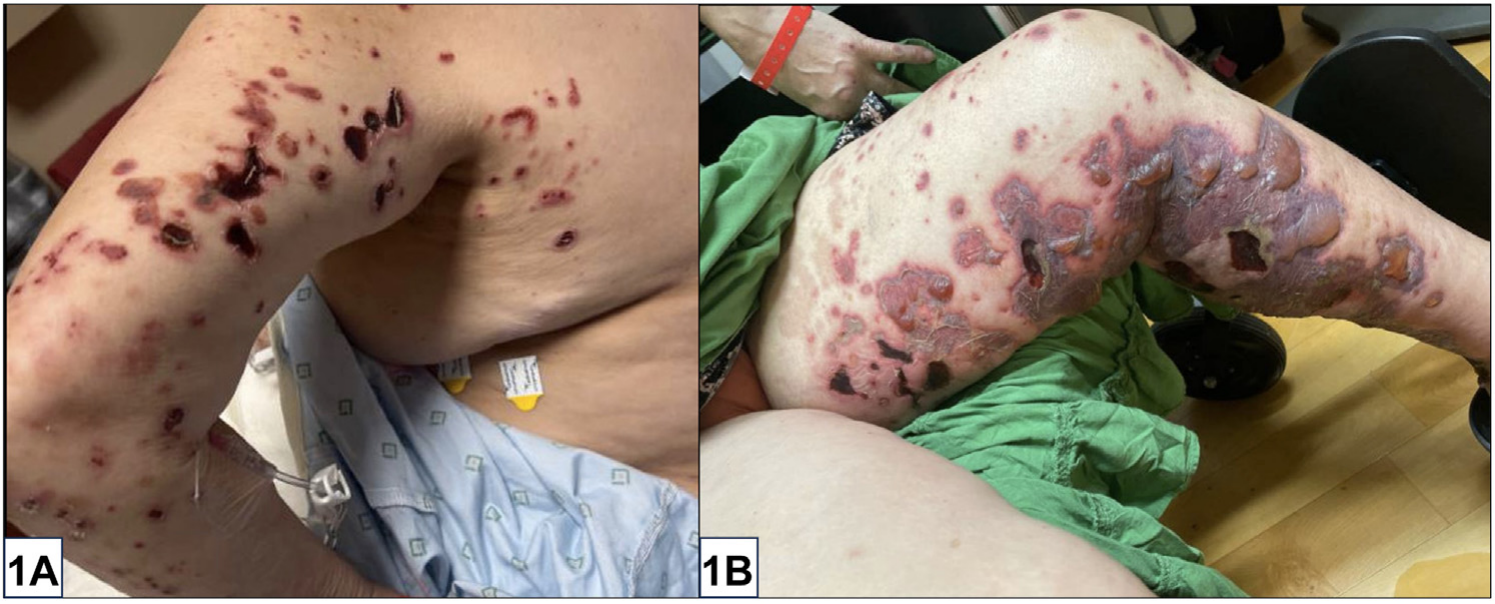

**Fig 2:**
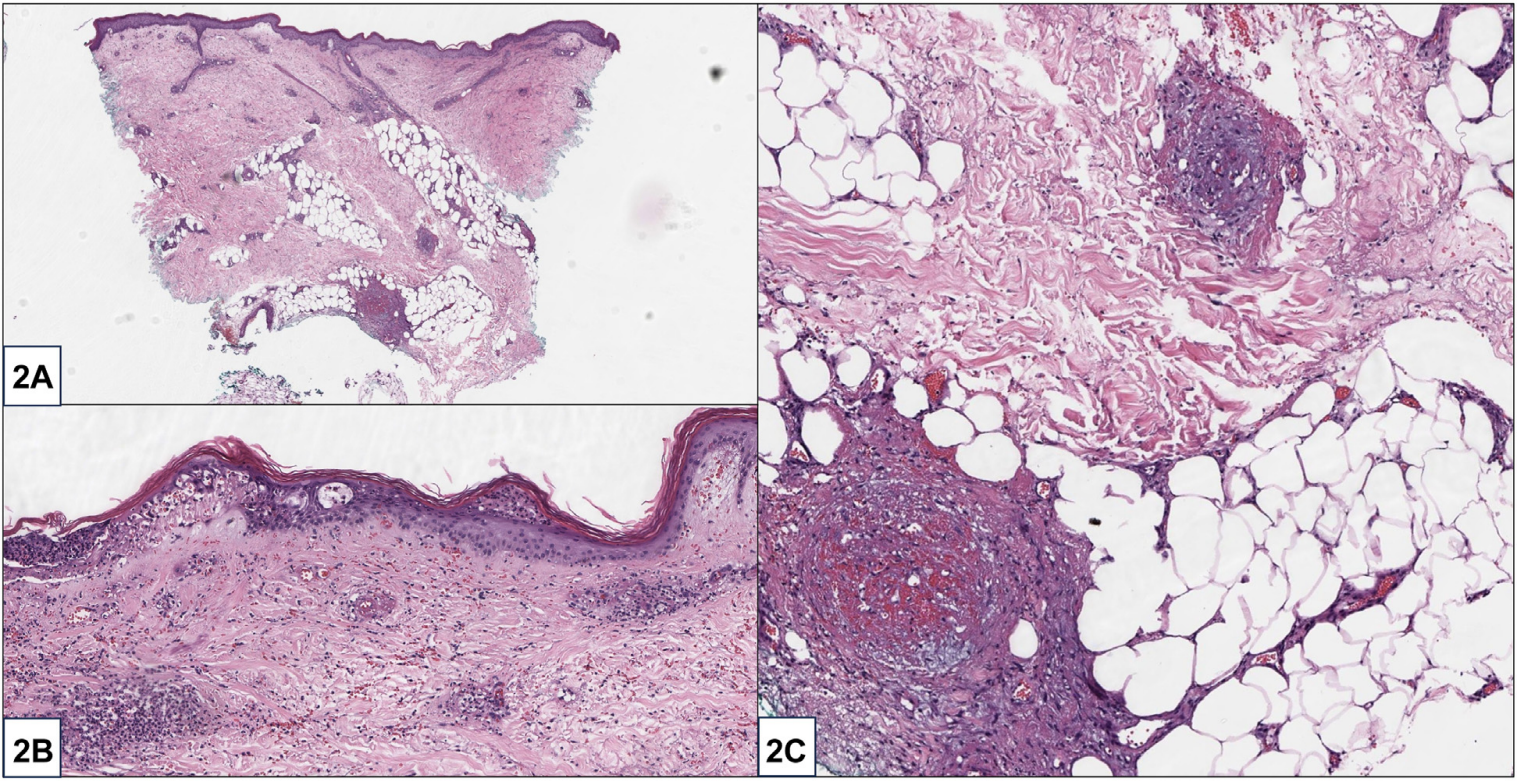

A 56-year-old woman with untreated polyarthritis presented with inflammatory retiform purpura, tense bullae, ulcers of the upper and lower extremities (Fig 1), numbness of the extremities, and new-onset right wrist drop. She denied fevers, lymphadenopathy, or unintentional weight loss. Initial labs were remarkable for a positive rheumatoid factor (RF) (41 U/ml) and anticyclic citrullinated peptide (>250 U/ml). Complement levels, antinuclear antibody, serum cryoglobulins, antineutrophil cytoplasmic antibody indirect immunofluorescence, antimyeloperoxidase, antiproteinase 3, and anti-Ro/La were all negative. X-rays of bilateral hands demonstrated extensive pancarpal inflammatory arthropathy without significant synovitis. Skin biopsy demonstrated small and medium vessel vasculitis with secondary fibrin thrombi (Fig 2).

**Question 1: Based on the clinical and laboratory data, what is the most likely diagnosis?**

A. Bullous leukocytoclastic vasculitis (LCV)
B. Polyarteritis nodosa (PAN)
C. Rheumatoid vasculitis (RV)
D. Cryoglobulinemic vasculitis
E. Paraneoplastic vasculitis

**Answers:**

A. Bullous LCV – Incorrect. While hemorrhagic bullae can also be observed in LCV, inflammatory retiform purpura are observed in medium vessel vasculitis rather than LCV. In addition, bullous LCV would not show medium vessel vasculitis on pathology.
B. PAN – Incorrect. PAN is excluded by the absence of nodules on examination and the presence of severe arthritis. PAN also demonstrates a focal medium vessel vasculitis on pathology.
C. RV – Correct. RV is a rare form of small- and medium-vessel vasculitis, occurring in 1% to 5% of patients with rheumatoid arthritis (RA).^1^ RV most often occurs in the setting of longstanding untreated seropositive RA due to the RF-mediated activation of complement and destruction of vessel walls.^2^ The skin is involved in 80% of patients, with common manifestations including purpura, ulcers, digital necrosis, and livedo reticularis.^3^^,^^4^ Histopathology frequently demonstrates thrombotic vasculitis affecting both small and medium vessels, a helpful but nonspecific feature.^3^
D. Cryoglobulinemic vasculitis – Incorrect. Cryoglobulinemic vasculitis can present with a positive RF, small and medium vessel vasculitis, and mixed serum cryoglobulins. However, anticyclic citrullinated peptide is highly specific for RV, and cryoglobulinemic vasculitis demonstrates vasculitis in combination with occlusion due to solid pink aggregates of cryoprotein rather than fibrin.
E. Paraneoplastic vasculitis – Incorrect. Retiform purpura and sensory changes can also be present in paraneoplastic vasculitis, but this is less likely based on the serologic evaluation and absence of constitutional symptoms.

**Question 2: Which of the following findings is the worst prognostic factor for patients with RV?**

A. Mononeuritis multiplex
B. Human leukocyte antigen (HLA) class I and II genotypes
C. Prior use of biologics for RA
D. Pancarpal arthropathy
E. Presence of Bywaters lesions

**Answers:**

A. Mononeuritis multiplex – Correct. Mononeuritis multiplex, which is sensory and motor deficits in 2 or more distinct nerve distributions, occurs in patients with RV due to occlusion and destruction of the vasa nervorum.^2^ The differential diagnosis of medium vessels vasculitis associated with mononeuritis multiplex is narrow and includes connective tissue disease (RA, systemic lupus erythematosus, Sjogren syndrome), PAN, antineutrophil cytoplasmic antibody vasculitis, mixed cryoglobulinemia, and paraneoplastic vasculitis.^3^ Because of this relatively narrow differential diagnosis, when present, mononeuritis multiplex is a useful clue for potential causes of vasculitis.
B. HLA class I and II genotypes – Incorrect. Male sex, smoking, rheumatoid nodules, longstanding disease, and HLA class I and II genotypes are associated with an increased risk of RV but are not associated with prognosis.^3^
C. Prior use of biologics for RA – Incorrect. Use of biologic and immunosuppressive treatments for RA reduces the risk of developing RV by reducing RF-mediated activation of complement and destruction of vessel walls.
D. Pancarpal arthropathy – incorrect. Pancarpal arthropathy is commonly seen in patients with severe RA. Although a history of severe RA increases the risk of developing RV, it does not directly impact prognosis.
E. Presence of Bywaters lesions – Incorrect. Bywaters lesions are small, asymptomatic, violaceous papules around the nails due to nailfold thrombosis that are associated with RV but are non-prognostic.

**Question 3: Which of the following is the most appropriate treatment for this patient?**

A. Etanercept
B. Secukinumab
C. Hydroxychloroquine
D. Rituximab
E. Methotrexate

**Answers:**

A. Etanercept – Incorrect. While tumor necrosis factor-α inhibitors have been used to treat RA, etanercept has been linked to an increased risk of LCV and is not the most appropriate treatment for severe RV.
B. Secukinumab – Incorrect. Secukinumab is an interleukin-17 inhibitor with US Food and Drug Administration approval for psoriasis and hidradenitis suppurativa.
C. Hydroxychloroquine – Incorrect. Mild-to-moderate cases of RV can be managed with combination hydroxychloroquine and topical steroids, but monotherapy with hydroxychloroquine is inadequate for severe disease.
D. Rituximab – Correct. While no standardized treatment algorithm for severe RV exists, several regimens have been reported, including pulse corticosteroids and cyclophosphamide either independently or in combination.^5^ 80 percent of patients with RV treated with rituximab experienced complete remission within 12 months.^5^ The patient in this case was treated with pulse methylprednisolone 500 mg intravenous daily for 3 days followed by prednisone 1 mg/kg/day and rituximab 1 g intravenous days 0 and 15.
E. Methotrexate – Incorrect. Although appropriate for mild RA with skin and peripheral nerve involvement, monotherapy with methotrexate is inadequate for severe RV.

## Conflicts of interest

None disclosed.
